# Longitudinal Myocardial Deformation Analysis of the Left Ventricle in Dogs with Leishmaniosis Investigated by Speckle-Tracking Echocardiography

**DOI:** 10.3390/pathogens15040370

**Published:** 2026-03-31

**Authors:** Alessandra Recchia, Antonella Colella, Maria Albrizio, Fabrizio Iarussi, Giovanni Romito, Aleksandra Domanjko Petrič, Paola Paradies

**Affiliations:** 1 Department of Precision and Regenerative Medicine and Ionian Area (DiMePRe-J)–Veterinary Section, University of Bari “Aldo Moro”, Strada Provinciale Casamassima km3, Valenzano, 70010 Bari, Italy; alessandra.recchia1@uniba.it (A.R.); antonella.colella@uniba.it (A.C.); maria.albrizio@uniba.it (M.A.); fabrizio.iarussi@uniba.it (F.I.); 2 Department of Veterinary Medical Sciences, University of Bologna, 40064 Ozzano dell’ Emilia, Italy; giovanni.romito2@unibo.it; 3 Veterinary Faculty, University of Ljubljana, 1000 Ljubljana, Slovenia; aleksandra.domanjko@vf.uni-lj.si

**Keywords:** dogs, leishmaniosis, left ventricular function, myocardial deformations, speckle tracking, strain

## Abstract

Inflammatory myocardial involvement has been reported in canine leishmaniosis (CanL); however, studies evaluating the degree of myocardial dysfunction in affected dogs are limited. This prospective study aimed to investigate myocardial involvement in dogs with CanL using conventional and speckle-tracking echocardiography (STE), focusing on the assessment of left ventricular systolic function and myocardial strain. Symptomatic, initially untreated dogs with a diagnosis of leishmaniosis and free from other vector-borne diseases or underlying heart diseases were enrolled (Leish group). Healthy dogs matched for age, body weight, breed, and sex were selected for the control group (C group). At the time of inclusion (T0) and at each follow-up, laboratory tests as well as conventional echocardiographic examination and STE were performed. For strain analysis, apical longitudinal long-axis 4-chamber, 3-chamber, and 2-chamber views were used (2C, 3C, 4C, respectively) to obtain the average global longitudinal strain (GLSAV), which is recognised to have the maximum reliability as an indicator of left ventricular dysfunction in humans. The software obtains GLSAV by averaging the longitudinal strain values from all left-ventricular segments derived from the multiple apical views. After enrolment, dogs were treated with a combination of meglumine and allopurinol and were monitored for six months. Clinical-pathological and echocardiographic data were collected at follow-up at 1, 3, and 6 months after the start of treatment (T1, T2, T3) and compared between the two study groups using appropriate statistical tests. Sixteen dogs composed the C group and nine dogs the Leish group. At T0, none of these dogs had abnormalities in cardiac auscultation, plasma cardiac troponin concentration was within the reference range, and standard echocardiographic examination excluded underlying cardiac diseases. The comparison between C and Leish groups did not show a statistically significant difference in any of the strain parameters analysed (GLSAV, GLS4C, GLS3C, GLS2C). Moreover, strain values in the Leish group did not change significantly over time. In conclusion, in this preliminary study on a limited population of dogs with leishmaniosis, both conventional echocardiography and STE failed to reveal clear changes suggestive of left ventricular systolic dysfunction secondary to possible myocarditis or as a consequence of the systemic disease in dogs with active leishmaniosis. However, further STE studies in larger cohorts of dogs with leishmaniosis are needed to confirm and expand our findings.

## 1. Introduction

Cardiovascular involvement in dogs with leishmaniosis have been recently reviewed [1]. Myocardial involvement in canine leishmaniosis (CanL) has been documented through post-mortem histopathological evaluations of myocardial samples from infected/diseased dogs [2,3,4] and by elevated levels of cardiac troponin I (cTnI) [5]. Such involvement occurs on an inflammatory basis, with predominantly lymphoplasmacytic inflammatory infiltrate [1]. The mechanisms inducing cardiac injury and myocarditis are still under discussion; in particular, *Leishmania* spp. amastigotes in the muscle may act as a trigger for the inflammatory response, or muscle damage could be related to immune-mediated mechanisms associated with the infection [6]. In fact, antigenic characterisation studies of infiltrating mononuclear cells and major histocompatibility complex (MHC) I and II class expression in myocarditis associated with *Leishmania infantum* infection in dogs provide evidence that, during leishmaniosis, myocarditis can occur with morphological and immunophenotypic patterns similar to canine myositis associated with *L. infantum* infection [7]. A study examining the hearts of 30 dogs infected with *L. infantum chagasi* [3] found myocardial lesions in all dogs despite the parasite only being found in the cardiac tissue of 20/30 dogs. More recently, in a study of 31 dogs whose cardiac tissue were examined, most showed cardiac injuries, but amastigotes were detected in only one dog [8]. Pericarditis associated with the presence of *Leishmania* spp. amastigotes in pericardial fluid have also been documented [9]. However, studies evaluating the degree of myocardial dysfunction in affected dogs are currently limited.

Speckle-tracking echocardiography (STE) is a technique developed for the study of myocardial deformations and function [10]; it is a semi-automatic technique independent of the angle of the ultrasound beam. Over the last ten years, two-dimensional STE (2D-STE) analysis has become increasingly important in human medicine and is also becoming a promising tool for assessing myocardial function in veterinary medicine. There is growing evidence that deformation parameters can be used as an indicator of myocardial function compared to conventional parameters for earlier diagnosis, as well as a possible prognostic indicator in both human and veterinary medicine [10]. In human medicine, the reference parameter is global longitudinal strain (GLS), according to the 2015 international guidelines [11]. The maximum reliability is recognised in the GLS value obtained from the average of the longitudinal strains obtained from the three different left apical scans (2C, 3C, 4C) representative of the entire ventricle (GLSAV) [12].

In veterinary medicine, longitudinal strain has been mostly measured on a single 4C scan, and no absolute normal ranges are available because breed, age group, scanning angle, and analysis software can influence the results [13,14,15]. Such variability makes it difficult to interpret strain data absolutely, which needs to be evaluated over time or in comparison with a control group.

We hypothesise that dogs affected by CanL present inflammatory involvement of the myocardium, which could result in altered myocardial deformations and consequent decreased cardiac function. This study is, therefore, aimed to longitudinally evaluate dogs with CanL before and after treatment using conventional echocardiography and 2D-STE and compare them at the time of diagnosis with clinically healthy dogs to identify whether a reduction in left ventricular longitudinal systolic function is associated with the disease.

## 2. Materials and Methods

Dogs with active clinical leishmaniosis (Leish group) were enrolled based on compatible clinical and laboratory findings, positivity on quantitative (ELISA, IFAT) or qualitative serological tests (rapid test) for *Leishmania* spp., and cytological lymph-nodal or bone marrow tests. Dogs had not received any treatment prior to inclusion and had tested negative for other vector-borne pathogens at the Snap test 4DxPlus, (Idexx Europe, Hoofddorp, The Netherlands), including *Dirofilaria immitis*, *Borrelia burgdorferi*, *Ehrlichia canis*, *Ehrlichia ewingii*, *Anaplasma phagocytophilum*, and *Anaplasma platys*. Animals with known heart diseases diagnosed before the diagnosis of leishmaniosis or with concomitant systemic diseases potentially responsible for cardiac alterations (e.g., diabetes mellitus, heartworm disease, hypoadrenocorticism, and hyperadrenocorticism) were also excluded from the study. The control group (C group) was created at a later stage based on age, body size, breed, and sex in order to obtain a homogeneous group comparable to the Leish group (Table 1). These control animals had to be in good clinical condition, have normal routine blood tests, and be negative on serology for *L. infantum* and at Snap test 4DxPlus (Idexx, Europe, Hoofddorp, The Netherlands).

At inclusion (T0), each dog from both groups underwent a medical history review, clinical examination, blood pressure measurement assessed by an oscillometric device (Suntech^®^ Vet30TM, SunTech Medical, Morrisville, NC, USA) and interpreted according to current guidelines [16], complete urinalysis with sediment evaluation and urine protein creatinine ratio (UPC), haemato-biochemical tests (complete blood count, biochemistry, electrophoresis), and complete conventional echocardiography (further details below). The latter was performed to rule out congenital or acquired heart diseases and to acquire standard echocardiographic parameters. In both groups, specific scans were also performed during the echocardiographic examination for further evaluation using the semi-automatic STE method (further details below). Moreover, in the Leish group, the serum concentration of cTnI was measured using chemiluminescence immunoassays [10] (range 0.0–0.08).

After inclusion, dogs were treated with a combination therapy protocol with allopurinol (10 mg/kg/os/bid for 6 months) and the first-line leishmanicidal meglumine antimoniate at 50 mg/kg/sc/bid for 30 days. Patients were re-evaluated in follow-ups at the end of their leishmanicidal therapy (T1), i.e., one month after T0, and again at 3 months (T2), and 6 months (T3) after T0. At each reassessment, all clinical and laboratory evaluations performed at T0 were repeated, as well as the evaluation of left ventricular systolic function using conventional echocardiography and 2D-STE. Proteinuria at each follow-up was assessed by a semi-quantitative method via the Combur test and the dogs were classified, in the absence of active sediment, as non-proteinuric vs. proteinuric: score 1 (30 mg/dL), 2 (100 mg/dL), 3 (550 mg/dL). UPC was repeated at T3 in all dogs.

At T3, all dogs underwent popliteal lymph-nodal sample collection for a cytological investigation on the presence of the *Leishmania* spp. amastigote.

The study was approved by the Ethics Committee for Veterinary and Zootechnical Clinical Studies of the Department of Precision and Regenerative Medicine–Ionian Area, with protocol no. 2001 of 20 May 2024.

### 2.1. Standard Echocardiographic Exam

The echocardiographic examination was performed on all dogs using an Esaote MyLabX75VET machine (Esaote, Firenze, Italy) and a 2–5 MHz cardiological probe, adjusting the frequency according to the size of the animal. The dogs were not sedated; trichotomy of the right and left cardiac areas was performed, and they were gently kept in lateral recumbency on a cardiological table, on the right and then on the left side [17]. The echocardiographic examination was performed with simultaneous single-lead ECG recording. Each dog underwent a complete echocardiographic mono- and two-dimensional examination (M- and B-mode, respectively), as well as an assessment of blood flows using spectral and colour Doppler, using the right parasternal long-axis 4- and 5-chamber views, the right parasternal short-axis views at the level of the papillary muscles and aortic root, the left apical 4- and 5-chambers views, and the cranial apical view. Left ventricular fractional shortening (FS%) was assessed using the M-mode at the level of papillary muscles using the right parasternal short-axis view, using the standard formula and considering as-normal values > 25% [18]. The left ventricular ejection fraction (EF%) was calculated both in M-mode, from the aforesaid view, and in B-mode from the right parasternal long-axis 4-chamber view using the Simpson method and considering as-normal values > 40% [18]. This last method and view were also used to calculate the end-diastolic and end-systolic left ventricular volumes, which were subsequently indexed for the body surface area (EDVI and ESVI, respectively). These two last parameters, when derived from M-mode measurements, were considered normal when ≤ 100 mL/m^2^ and 30 mL/m^2^, respectively [18,19].

### 2.2. Speckle-Tracking Echocardiography

To assess left ventricular myocardial deformities, the STE method was used to acquire longitudinal strain (GLS) parameters. For the STE examination, 3 specific left views were taken following the conventional examination, using the same machine and probe. For strain analysis, video clips lasting three cardiac cycles were acquired and used for offline post-processing analysis. Three views were used, namely the left apical 4-chamber (4C), 3-chamber (alax or 3C), and 2-chamber (2C) views, as previously described in humans, and recently in dogs [15,20,21]. Left ventricular myocardial strain was analysed using dedicated software installed in the machine (XStrain^TM^LV, version MYEVO2024). The machine automatically defines the endocardial borders after defining three points corresponding to the mitral valve and the cardiac apex (semi-AUTO strain) and recognises the left ventricular wall. The borders defined by the machine can be optimised using the manual method if needed (manual tracing). The software then processes the acquisitions by automatically dividing the left ventricle into six segments, measures the endocardial strain of each segment, expressed as a percentage, and calculates the global strain for each longitudinal scan (GLS2C, GLS3C, GLS4C). From these, it derives the total average global longitudinal strain value (GLSAV or mean longitudinal global strain).

### 2.3. Statistical Analysis

Statistical analysis was performed using IBM SPSS Statistics 19 software. Nonparametric tests were utilised because of the limited number of subjects under investigation.

In the longitudinal study, the values of haematocrit, haemoglobin, platelets, urea, creatinine, phosphorus, albumin, albumin/globulin ratio, total protein, gamma globulin, urinary specific gravity, proteinuria score, systolic blood pressure, diastolic blood pressure, echocardiographic conventional EF (M-mode), FS (M-mode), EF (B-mode), FS (B-mode), ESVI, EDVI, and STE values GLS2C, GLS3C, GLS4C, and GLSAV, at different time points were analysed using the nonparametric Friedman test; Wilcoxon’s test was employed post hoc for pairwise comparisons and for echocardiographic measurements, and *p*-values were adjusted according to Bonferroni for multiple comparisons. Values of *p* < 0.05 were considered significant. Selected parameters were presented as median and IQR (interquartile range) by boxplot graphs. The echocardiographic strain parameters of the Leish group at T0 and the C group were statistically compared using Friedman’s test. Values of *p* < 0.05 were considered statistically significant.

## 3. Results

Nine dogs with clinical leishmaniosis (Leish dogs) were included in the longitudinal study. In particular, there were three mixed breed, three German Shepherd, one Amstaff, one Great Dane, and one Presa Canario, aged from 3 to 10 years (median 5 years), weighing from 9 to 60.2 kg (median 30.35). The most common clinical signs and presenting complaints at T0 were: dermatological signs n = 7 (78%), lymphadenopathy n = 7 (78%), weight loss n = 3 (33%), disorexia n = 2 (22%), and lethargy n = 2 (22%). Other signs were hyperthermia, unsteady walking, and uveitis referred in individual dogs. According to the LeishVet guidelines [22], dogs were classified as Stage II b (n = 5), Stage III (n = 3), and Stage IV IRIS III (n = 1).

**Figure 1:**
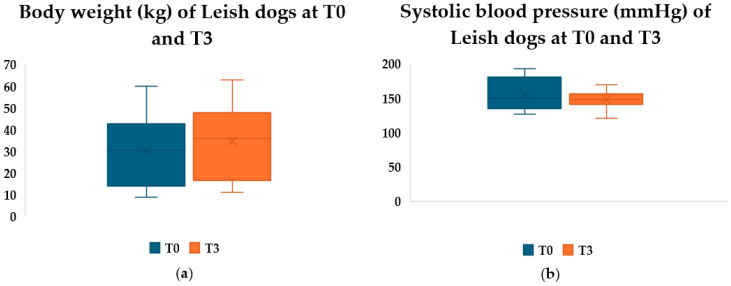
(**a**) Boxplot visualisation of body weight of Leish group dogs at T0 and T3; (**b**) Boxplot visualisation of systolic blood pressure of Leish group dogs at T0 and T3.

All dogs showed clinical improvement at T1, and a complete reversal of clinical signs was reached at T3 in all dogs. Body weight progressively increased in all dogs from T0 to T3 (Figure 1). Systolic blood pressure at T0 and T3 are also reported in Figure 1.

Laboratory data at T0 and T3 are shown in Table 2.

Only three dogs showed normocytic normochromic anaemia at T0 with remission at T1. In general, HCT of all dogs showed an upward trend between T0 and T3, except for one dog, for which the HCT tended to decrease again at T3. All dogs showed, at T0, an altered albumin/globulin ratio (ALB/GLOB < 0.66) due to an increase in the gamma serum protein fraction; however, only three dogs had increased total protein. The gamma fraction of protein % showed a downward trend between T0 and T3 in all dogs except one. At inclusion, only one dog showed alterations indicative of hyperazotemia with proteinuria and systemic hypertension, that improved after treatment. Urinary protein score decreased after treatment in 7/9 dogs (78%) and did not worsen in any dog. UPC reduced significantly from T0 to T3 (*p* < 0.05). The cTnI values were within the reference range (0–0.08 ng/mL) in all dogs except for two, showing a very mild increase (0.11 and 0.16 ng/mL). Statistical analysis using the Friedman and the Wilcoxon’s post hoc test showed significant differences between T0 and the follow-ups for HCT (*p* < 0.05), haemoglobin (HGB) (*p* < 0.05), total protein (TP) (*p* < 0.05), albumin (*p* < 0.01), ALB/GLOB (*p* < 0.01), and gamma globulin fraction (*p* < 0.01). The data are shown in Table 3. No significant differences were revealed over time for the other studied variables: platelets, urea, creatinine, phosphorus, systolic blood pressure, diastolic blood pressure, urine specific gravity, and urinary protein score.

**Figure 2:**
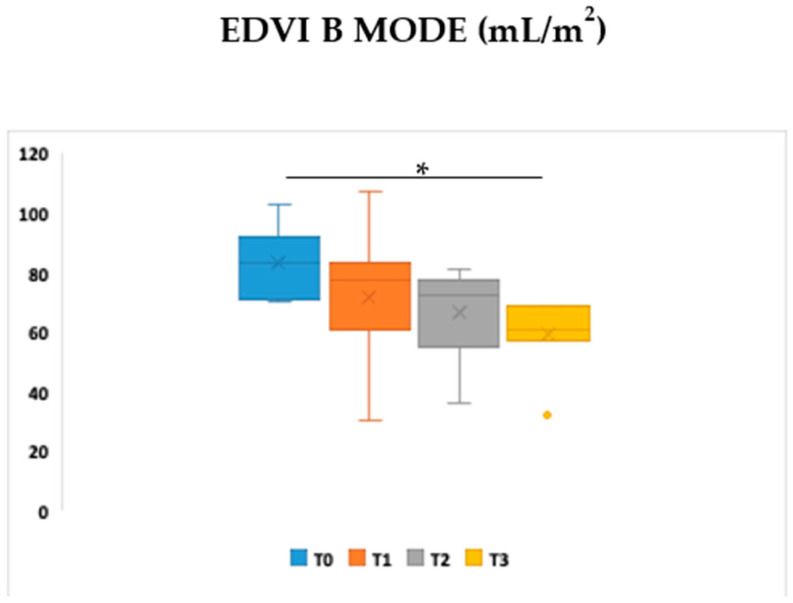
Boxplot visualisation of EDVI measured at different time points in the Leish group. Friedman’s nonparametric test was applied to evaluate statistically significant differences. Wilcoxon’s post hoc test was used for pairwise comparisons, and *p*-values were adjusted according to Bonferroni for multiple comparisons. * = *p* < 0.05.

The standard echocardiographic examination at T0 did not reveal any underlying cardiac abnormalities in any animal. Furthermore, absence of rhythm abnormalities was registered on the ECG monitoring performed during the echocardiogram. Statistical analysis of conventional echocardiographic data at different time points revealed significance only for EDVI (T0 *vs.* T3 *p* < 0.05) (Table 4, Figure 2). No significance was found for the other conventional functional parameters. Beyond the statistics, when evaluating individual patients, in a single dog, some parameters were abnormal at T0 (FS 23%, ESVI 49 mL/m^2^, EDVI 103 mL/m^2^). In this dog, an echocardiography 6 months after treatment with the standard antimoniate and allopurinol protocol showed a normalisation of FS%, ESVI, and EDVI.

**Figure 3:**
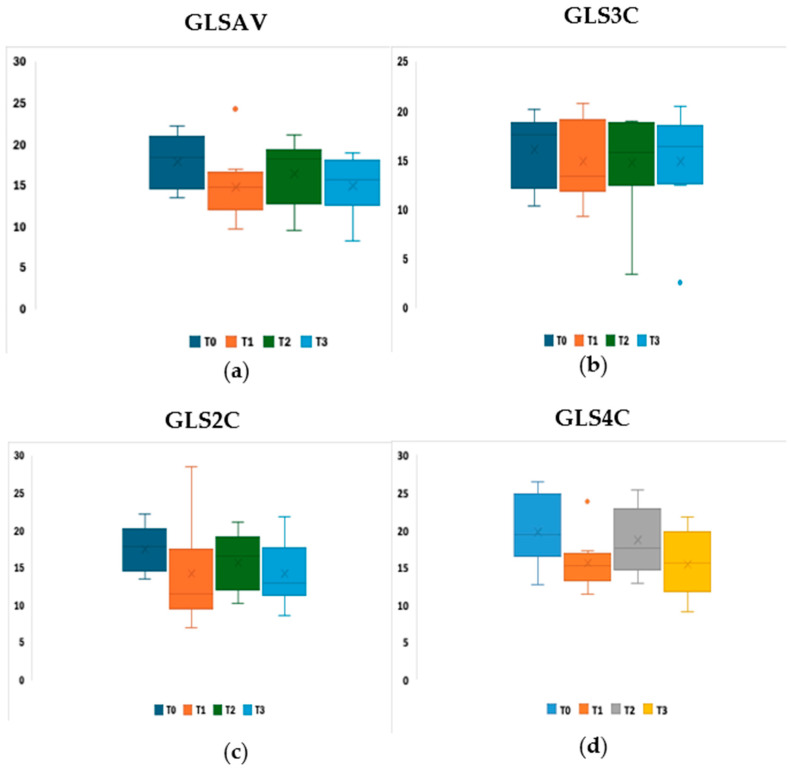
Boxplot visualisation of longitudinal myocardial strain values in the Leish group, divided by echocardiographic plane (**a**) four-chamber (GLS4C); (**b**) two-chamber (GLS2C); (**c**) three-chamber (GLS3C); (**d**) average global longitudinal strain (GLSAV). No statistically significant difference between T2 and T3 (Friedman’s nonparametric test and the Wilcoxon’s post hoc test for pairwise comparisons were applied; *p*-values adjusted using the Bonferroni corrections).

With regard to strain values, no statistical significance was identified in the Leish group over time for GLSAV, GLS4C, GLS3C, and GLS2C (Figure 3, Table 5).

For the C group, 16 dogs aged from 2 to 9 years (median 5 years) were selected with breed, weight, and sex characteristics kept as homogeneous as possible when compared to the Leish group, as already reported in Table 1.

In the C group, all conventional echocardiographic parameters were within normal limits. The evaluation of STE data in the C group allowed us to identify the minimum and maximum reference ranges for each strain using the same machine, software, and operator as in the Leish group. In particular, for GLSAV, the values ranged from −15.43 to −23.43. The data are shown in Table 6.

**Figure 4:**
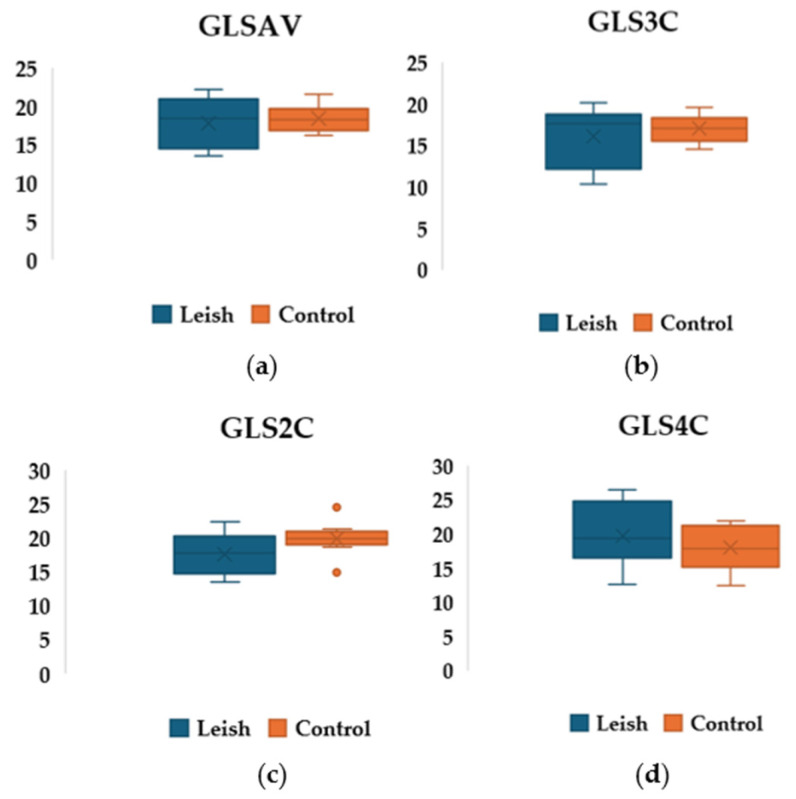
Boxplot representation of longitudinal myocardial strain values (**a**) GLS4C; (**b**) GLS3C; (**c**) GLS2C; (**d**) GLSAV. Data, recorded at T0 in Leish and C groups, were compared by the Friedman’s nonparametric test. No statistically significant difference was found.

Statistical analysis comparing strain parameters between the Leish group and the C group at T0 showed no statistically significant differences. In particular, GLSAV was comparable for the two groups (Table 7 and Figure 4).

## 4. Discussion

This study, that aimed to longitudinally evaluate dogs with leishmaniosis before and after treatment using conventional echocardiography and STE and compare them at the time of diagnosis with clinically healthy dogs, failed to identify left ventricular functional alterations associated with the disease.

Our findings should be interpreted in light of the previous scientific literature. It is well-known that cardiovascular involvement can occur in dogs with leishmaniosis, as demonstrated by histopathological evidence of myocardial inflammatory lymphoplasmacytic infiltrates [1]. Moreover, leishmaniosis may also cause reduced cardiac contractility and increased cTnI concentrations through cytokine-mediated inflammatory mechanisms [1,5,6]. However, documentation of active myocarditis or associated clinical signs is rare, and detection of the parasite in the heart by immunohistochemistry has been reported only in a few studies [23,24]. Similarly, with conventional echocardiographic examinations, signs of cardiac dysfunction have been documented only in a few dogs with severe disease [25,26] or dogs coinfected with *Ehrlichia canis* [27]. Additionally, in these studies, it cannot be confirmed that leishmaniosis alone was the sole cause of echocardiographic alterations, or if other systemic diseases had an effect on myocardial function [1].

In general, all dogs from this study responded well to therapy with significant improvement, recorded already at T1, in both clinical and haemato-biochemical parameters, as previously reported [28,29,30]. All of them reached clinical disease remission and a negative result at lymph-nodal cytological examination at T3. None of our patients experienced adverse effects or renal repercussions from treatment. Instead, a general reduction in proteinuria was recorded, as previously reported [30,31,32]. Efficacy of treatment for *Leishmania* spp. is based on clinical assessments (i.e., remission or reduction in symptoms, increase in body weight), haemato-biochemical assessments, and renal function assessments [29].

During clinical examination of the dogs in the Leish group at T0, no animal showed murmurs or arrhythmias, and the standard echocardiographic examination did not reveal any underlying congenital heart disease or valvular disease. Moreover, no signs suggestive of myocarditis (e.g., left ventricular global/segmental systolic dysfunction associated with eccentric hypertrophy, focal/diffuse myocardial thickening with echogenicity alterations due to cellular infiltration and interstitial edema, pericardial effusion, or arrhythmias [33,34,35,36]) were found.

With specific regard to the most commonly used direct/indirect indices of left ventricular systolic function (namely, FS%, EF%, ESVI, and EDVI [37,38]), it is important to underline that their use did not allow the recognition of obvious systolic deficits in dogs with leishmaniosis at enrolment (T0). Statistical analysis revealed only a significant difference between T0 and T3 for EDVI, indicating a reduction in diastolic volume potentially associated with functional improvement after treatment.

At the same time, it should be considered that standard echocardiography may appear normal in patients with myocarditis (confirmed by endomyocardial biopsy), especially in chronic subclinical forms [39]. For this reason, a method such as STE has proven useful in providing a more accurate representation of contractility and regional dysfunctions that cannot otherwise be assessed using routine parameters [40], enabling earlier diagnosis of myocarditis [38].

Although, in veterinary medicine, global strain is usually assessed along three axes (longitudinal, radial, and circumferential), the decision to evaluate only global longitudinal strains (GLS) in the present study was based on its documented higher reproducibility compared with the other two parameters in human medicine [41,42]. In fact, longitudinal deformation is considered the best indicator of left ventricular dysfunction and is considered more sensitive than EF% in people [43]. More specifically, in humans, the maximum reliability is recognised in the GLS value obtained from the average of the longitudinal strains obtained from the three different left apical scans (2C, 3C, 4C), representative of the entire ventricle (GLSAV) [12].

In veterinary literature, absolute parameters of normal longitudinal strain in dogs are scarce and quite variable and usually obtained from a single 4-chamber scan (GLS4C). Only combining data from studies conducted by different authors using different software and equipment on healthy dogs [13,41,44,45], we extrapolated a normal reference range for GLS4C ranging from −12 to −25. The most extensive data on non-purebred dogs are those of Wess et al. (2011) [46], who reported a range of −16 ± 4 out of 100 dogs.

To date, only three studies [15,20,21] have used the three standard apical views derived from humans, as in our work, to assess GLSAV. The study by Hertzsch and Wess (2023) [15], conducted on 120 healthy Doberman Pinschers, reported a normal GLSAV range between −24.2 and −16.5, using AutoSTRAIN/CPA software with a Philips EPIQ 7c system. The most recent study by Mogensen et al. (2025) [21] on only 10 healthy dogs of different breeds, reported a GLSAV of −20.04 ± 2.88 with 2D-strain x software and −17.67 ± 1.61 with GE Healthcare’s automatic function (AFI) software. Meanwhile, Wang et al., 2025 [20] evaluated radiation-induced heart disease in two group of Beagles (18 dogs of the irradiation group and 18 dogs of the control group) by 2D-STE, using an Echo PAC software (2D-STE analysis software—GE Healthcare). At baseline, before irradiation, both groups showed a GLSAV of 20.02 ± 0.9 (control group) and 19.92 ± 0.65 (irradiation group).

Therefore, the GLSAV results acquired by our C group, with a range varying between −15 and −23, do not differ greatly from those reported in the literature, despite being obtained with different software and equipment.

It should be noted that, although the longitudinal strain is a negative strain, when referring to strain variations, the absolute value of the number is considered, as indicated by the “Task force for standardising deformation imaging” [11].

In the Leish group at T0 (corresponding to the symptomatic phase of the disease), on the basis of previous studies on myocarditis [47], a reduction in GLSAV would have been expected as a potential indicator of myocardial inflammation associated with leishmaniosis and consequent impairment of left ventricular function. Furthermore, a measurable difference between T0 and post-treatment follow-up evaluations would have been expected, indicative of improved myocardial performance after treatment in the case of subclinical myocarditis and cardiac injury associated with leishmaniosis.

A reduced speckle-tracking-derived longitudinal endocardial strain at T0 and a functional improvement after treatment could also be expected as a consequence of the systemic inflammatory condition inducing myocardial depression, as previously reported in dogs with systemic inflammatory response syndrome [48].

Since it was not possible to rely on absolute GLSAV values clearly suggestive of myocardial deformation, the analysis focused on evaluating potential statistically significant differences in myocardial deformation between T0 and subsequent time points (dogs with active disease vs. post-treated dogs). Overall, no significant reduction in strain was observed at T0 compared with T1, T2, and T3 that would indicate myocardial involvement detectable by strain analysis in the diseased dogs.

It should also be noted that cTnI was not altered in dogs in the Leish group at T0 (except for two with a very mild increase), unlike the results of a previous work where 40% of dogs with leishmaniosis showed cTnI increase [5]. The lack of abnormal cTnI supports that these dogs, despite being affected by CanL, did not have active myocardial inflammation, thus justifying the absence of strain alterations. It would be interesting to test, in the future, dogs affected by CanL with elevated cTnI values using STE. It is known that cTnI could also increase during systemic inflammatory conditions [49].

The comparison between the nine dogs affected by clinical leishmaniosis and the sixteen healthy dogs proved to be fundamental in giving meaning to the strain values measured in the study. Measuring GLSAV in the healthy dogs of the C group, using the same ultrasound machine, the same probe, and the same operator allowed us to further assess that our dogs with leishmaniosis, based on the evaluation of myocardial deformations, maintain good ventricular function despite systemic infection. It is noteworthy that, to the author’s knowledge, this is the first work using this specific software and machine, and specific reference ranges for the measurements obtained with the specific equipment used in our study were not available in the literature.

The present study is limited by the small sample size, the heterogeneity of clinical stages of Leish group dogs at inclusion, and the absence of endomyocardial biopsies (which represent the gold standard for the diagnosis of ongoing myocarditis). The study also has other limitations, such as focusing solely on GLSAV without considering radial or circumferential strain. At the same time, it should be noted that focusing on GLSAV provided a more stable and less operator-dependent parameter, reducing the complexity of the analysis and facilitating the replicability of the method in real clinical settings. Another technical limitation of the method is the need to obtain excellent scans for the machine to recognise the ventricular wall. Indeed, as this is a semi-automatic system that uses artificial intelligence, a scan that is not perfectly reproducible will be considered unacceptable, and the machine will not allow processing and analysis. The manual option to optimise the endocardial profile only allows minor adjustments to correct scans. However, this procedure is quite time-demanding, which may limit its use in daily clinical practice.

## 5. Conclusions

In the present study sample, echocardiography, both conventional and STE, did not reveal any changes attributable to myocardial alterations suggestive of myocarditis in active leishmaniosis. Given the small sample size, it cannot be ruled out that myocardial involvement was truly absent in these animals or that, as these were subclinical forms, such involvement was not detectable by the echocardiographic methods we used. Further studies, ideally involving a large population of affected dogs in which echocardiographic data are compared with histological examination obtained through endomyocardial biopsy, are needed to validate and expand our preliminary findings.

## Figures and Tables

**Table 1:** Differences in age, body weight, sex, and mixed vs. breed dogs between Control (C group) and Leish dogs. Data of age and body weight were analysed using the Mann–Whitney test, while sex and mixed/breed were evaluated by chi-square test. Values are reported as median and IQR, and as percentages for sex and breed.

Variable	Leish Group (n = 9)	C Group (n = 16)	*p*-Value
Age (years) median (IQR)	5 (2.62)	5 (3)	0.545
Body weight (kg) median (IQR)	30.35 (18.4)	27.15 (22.85)	0.821
Sex (male %)	55.55	56.25	1
Breed (mixed %)	33.3	50	1

**Table 2:** Laboratory data of the Leish group at T0 and T3.

	HCT (%)	HGB (g/dL)	PLT (1000/µL)	BUN (mg/dL)	CREA (mg/dL)	P (mg/dL)	ALB (%)	ALB/ GLOB	TP (g/dL)	γ (%)	Cardiac Troponin I (ng/mL)	USG	UPC
RI	(38.0–54.0)	(14.0–19.5)	(180–450)	(15–50)	(0.70–1.4)	(2.8–4.7)	(49.0–60)	(0.70–1.4)	(5.7–7.3)	(6.4–14.5)	(0–0.08)	(1030–1065)	
T0	37.7 (10.8)	12.5 (3.6)	234 (171)	33 (26)	0.93 (0.31)	4.1 (0.8)	28.4 (16.8)	0.4 (0.33)	7.2 (3.1)	29.9 (19.1)	(0.02–0.04)	1034 (17)	0.9 (0.6)
T3	43.8 (3.2)	14.1 (3.4)	200 (95)	32 (7)	1.04 (0.26)	4.1 (0.5)	43 (8.1)	0.79 (0.27)	7 (0.7)	20 (8)	-	1025 (27)	0.5 (1)

RI: range interval; HCT: haematocrit; HGB: hemoglobulin; PLT: platelets; BUN: blood urea nitrogen; CREA: creatinine; P: phosphorus; ALB: albumin; ALB/GLOB: albumin-to-globulin ratio; TP: total proteins; γ: gamma globulins; USG: urine specific gravity. Values are expressed as median and IQR.

**Table 3:** Over-time statistically significant laboratory results of the longitudinal study on Leish group.

	T0 *vs.* T1	T0 *vs.* T2	T0 *vs.* T3	T1 *vs.* T2	T1 *vs.* T3	T2 *vs.* T3
HCT (%)	37.5 (10.8); 43.8 (6) *p* = 0.008 *	37.5 (10.8); 42.2 (4) *p* = 0.008 *	37.5 (10.8); 43.8 (3.2) *p* = 0.028 *	43.8 (6); 42.2 (4) *p* = 0.248	43.8 (6); 43.8 (3.2) *p* = 0.352	42.2 (4); 43.8 (3.2) *p* = 0.398
HGB MEDIAN (IQR)	12.5 (3.6); 14.4 (2) *p* = 0.011 *	12.5 (3.6); 14.6 (1.4) *p* = 0.011 *	12.5 (3.6); 14.1 (3.4) *p* = 0.011 *	14.4 (2); 14.6 (1.4) *p* = 0.207	14.4 (2); 14.1 (3.4) *p* = 0.085	14.4 (2); 14.1 (3.4) *p* = 0.553
ALB MEDIAN (IQR)	28.4 (16.8); 38.9 (17.4) *p* = 0.011 *	28.4 (16.8); 41.8 (14.7) *p* = 0.021 *	28.4 (16.8); 43 (8.1) *p* = 0.036 *	38.9 (17.4); 41.8 (14.7) *p* = 0.139	38.9 (17.4); 43 (8.1) *p* = 0.208	41.8 (14.7); 43 (8.1) *p* = 0.575
ALB/GLOB MEDIAN (IQR)	0.4 (0.33); 0.6 (0.39) *p* = 0.015 *	0.4 (0.33); 0.72 (0.4) *p* = 0.021 *	0.4 (0.33); 0.79 (0.27) *p* = 0.021 *	0.6 (0.39); 0.72 (0.4) *p* = 0.110	0.6 (0.39); 0.79 (0.27) *p* = 0.173	0.72 (0.4); 0.79 (0.27) *p* = 0.944
TP MEDIAN (IQR)	7.4 (3.1); 7.2 (1.3) *p* = 0.011 *	7.4 (3.1); 7.2 (1) *p* = 0.051 *	7.4 (3.1); 7 (0.7) *p* = 0.021 *	7.2 (1.3); 7.2 (1) *p* = 0.123	7.2 (1.3); 7 (0.7) *p* = 0.050	7.2 (1); 7 (0.7) *p* = 0.288
ɣ% MEDIAN (IQR)	29.9 (19.1); 23.2 (18) *p* = 0.008 *	29.9 (19.1); 19.3 (9) *p* = 0.008 *	29.9 (19.1); 20 (8) *p* = 0.015	23.2 (18); 19.3 (9) *p* = 0.028 *	23.2 (18); 20 (8) *p* = 0.173	19.3 (9); 20 (8) *p* = 0.374

Data were analysed by Friedman’s nonparametric test; the Wilcoxon’s test was employed as post hoc for pairwise comparisons. *p* = observed significance level. * *p* < 0.05—significant difference, *p* < 0.01—highly significant difference, *vs.* = versus.

**Table 4:** Friedman’s test was employed to compare conventional echocardiographic parameters within the Leish group at the different time points.

	T0	T1	T2	T3	Friedman’s *p*-Value
FS %	34 (7)	32 (9)	33 (2)	29 (8)	*p* = 0.546
EF % (M-MODE)	61 (10)	54 (15)	61 (9)	55 (8)	*p* = 0.386
EF % (SIMPSON)	51 (13)	52 (8)	52 (17)	48 (15)	*p* = 0.254
ESVI	42.7 (20)	36.2 (18.8)	31.4 (8.9)	30.9 (8.1)	*p* = 0.269
EDVI	83 (20.9) ^a^	77.5 (19.2)	72.6 (21)	60.8 (10.6) ^b^	*p* = 0.004

FS: left ventricular fractional shortening; EF: left ventricular ejection fraction; ESVI: end-systolic left ventricular volumes indexed for the body surface area; EDVI: the end-diastolic indexed for the body surface area. Values are expressed as median and IQR; obtained *p*-values are shown. The post hoc test for pairwise comparisons was only applied for the EDVI parameter and ^a^, ^b^ *p* = 0.048.

**Table 5:** Friedman’s test was employed to compare strain parameters, expressed as median and IQR, within the Leish group at the different time points. Obtained *p*-values are shown.

	T0	T1	T2	T3	Friedman’s *p*-Value
GLS2C	−17.86 (4.27)	−11.53 (6.48)	−16.67 (6.24)	−13.07 (3)	*p* = 0.204
GLS3C	−17.68 (5.84)	−13.49 (6.06)	−15.89 (5.72)	−16.5 (5.46)	*p* = 0.954
GLS4C	−19.42 (6.1)	−15.28 (2.97)	−17.59 (5.78)	−15.6 (5.98)	*p* = 0.051
GLSAV	−18.52 (5.55)	−14.87 (4.4)	−18.21 (5.73)	−15.81 (4.42)	*p* = 0.115

**Table 6:** Range, median, and IQR of longitudinal myocardial strain values measured in dogs of the C group, reported for each echocardiographic plane (4C, 2C, 3C) and for the GLSAV.

C Group	Min (%)	Max (%)	Median	IQR
GLS4C	−12.5	−25.6	−19.29	5.31
GLS3C	−10.98	−21.91	−17.13	4.68
GLS2C	−13.05	−25.97	−19.97	4.58
GLSAV	−15.43	−23.43	−18.98	3.64

**Table 7:** Friedman’s test was employed to compare strain parameters, expressed as median and IQR, between Leish and C group at T0. Obtained *p*-values are shown.

	Leish vs. Control
GLS2C	17.86 (4.27); 20.01 (1.08); *p* = 0.085
GLS3C	17.68 (5.84); 17.1 (2.05); *p* = 0.965
GLS4C	19.42 (6.10); 17.90 (4.40); *p* = 0.627
GLSAV	18.52 (5.55); 18.32 (1.85); *p* = 0.79

## Data Availability

Data (Excel files, full exams) supporting reported results are available from the corresponding author.

## Abbreviations

The following abbreviations are used in this manuscript:

GLSAV	Average Global Longitudinal Strain
2D-STE	Two-Dimensional Speckle-Tracking Echocardiography
CanL	Canine Leishmaniosis
cTnI	Cardiac Troponin I
GLS	Global Longitudinal Strain
GLS2C	2-Chamber Longitudinal Strain
GLS3C	3-Chamber Longitudinal Strain
GLS4C	4-Chamber Longitudinal Strain
EF	Ejection Fraction
FS	Fractional Shortening
EDVI	End-Diastolic Volume Index
ESVI	End-Systolic Volume Index
HCT	Haematocrit
ALB/GLOB	Albumin to Globulin Ratio
